# QT interval dynamics in patients with ST-elevation MI

**DOI:** 10.3389/fcvm.2022.1056456

**Published:** 2023-01-06

**Authors:** Tomer Mann, Amit Moses, Anastasiea Yesaulov, Aviram Hochstadt, Yoav Granot, Raphael Rosso, Yacov Shacham, Ehud Chorin

**Affiliations:** Department of Cardiology, Tel Aviv Sourasky Medical Center and Sackler School of Medicine, Tel Aviv University, Tel Aviv, Israel

**Keywords:** QT, STEMI, dynamics, all-cause mortality, gender

## Abstract

**Background:**

An association between excessively prolonged QT and ventricular arrhythmia in patients with ST-elevation myocardial infarction has been described; however, the QT dynamics, characterization, and long-term predictive value are not well known.

**Objective:**

To characterize QT interval dynamics in patients undergoing ST elevation myocardial infarction (STEMI) and determine its association with mortality.

**Methods:**

A retrospective analysis of 4,936 consecutive patients, hospitalized for STEMI between 01/2013–12/2021. Patients with less than three electrocardiograms (ECGs) during index hospitalization were excluded. Baseline demographics, cardiovascular history, clinical risk factors, treatment measures, laboratory results, and mortality data were retrieved from the hospital’s electronic medical records.

**Results:**

We included 1,054 patients and 5,021 ECGs in our cohort with a median follow-up of 6 years [interquartile range (IQR) 4.3–7.4 years]. The QT was longer in women in comparison to men (428.6 ms ± 33.4 versus 419.8 ms ± 32.52, *P*-value = 0.001). QT prolongation was greater in females, elderly patients, and patients with STEMI caused by occlusion of the left anterior descending (LAD) coronary artery. We determined QT cutoff to be 445 ms. This value of QT divided our cohort upon arrival into a long QT group (217 patients, 26% of the cohort) and a “normal” QT group (835 patients, 74% of the cohort). The long QT group experienced an increase in combined short and long terms all-cause mortality. The QT upon arrival, on day 2 of hospitalization, and before discharge from the hospital, correlated with long-term mortality.

**Conclusion:**

QT duration is often prolonged during STEMI; this prolongation is associated with increased mortality and adverse events. Gender is an important mediator of QT dynamics.

## Introduction

The corrected QT interval (QT) is a valuable clinical tool to identify patients with a high risk of developing ventricular arrhythmias and sudden cardiac death (SCD) (1, 2). The QT interval is also useful for censoring adverse effects of pharmacological agents known to cause QT prolongation and thus place patients at higher risk for SCD (3). Fascinatingly, during the past few years evidence has suggested a prognostic role in the QT interval amongst critically ill patients (4). The period following acute coronary syndrome is often complicated by the life-threatening arrhythmias, sometimes correlated with prolonged QT intervals. Patients who continue to have a QT interval prolongation on their electrocardiograms (ECGs) post-acute coronary syndrome are at a higher risk for SCD (5–10). A correlation between extremely prolonged QT intervals and torsade de Pointes (TdP) in patients recovering from myocardial infarction (MI) was established by Viskin et al. and its incidence was assessed to be 1.8% (11).

The QT interval prolongation is the earliest event during ischemia (12). Abnormal QT interval prolongation has been reported in patients with ST elevation myocardial infarction (7–10) or unstable angina (13). Furthermore, QT has been found to be an independent predictor of arrhythmic death following acute MI (5, 14). The ACTION trial showed that a QT interval longer than 430 milliseconds in patients with coronary artery disease (CAD), was a predictor of death and was found to be comparable to 3-vessel disease (15); nevertheless, it was accomplished in patients with stable CAD. The QT interval prolongation returned to normal values 48 h after myocardial revascularization (16). The normalization of QT interval in patients who underwent angioplasty is speculated to be a marker of adequate reperfusion (17).

The objective of the current study is to describe QT dynamics among patients undergoing ST elevation myocardial infarction (STEMI) and define its association with short and long-term mortality, and to refine the surveillance of the QT by establishing timeline-specific high-risk QT cutoffs. This may provide valuable prognostic information and may guide initial triage decisions among STEMI patients and may help to identify high-risk patients.

## Materials and methods

### Study population

Patients admitted from January 2013 to December 2021 to the Cardiac Intensive Care Unit at the Tel Aviv Sourasky Medical Center with a diagnosis of acute STEMI were retrospectively identified (4,936 consecutive patients). We excluded patients with less than three ECG recordings during the index hospitalization (Supplementary Figure 1). All patients included in our study were treated with primary percutaneous coronary intervention (PCI). Baseline demographics, cardiovascular history, clinical risk factors, treatment measures, laboratory results, and mortality data were retrieved from the hospital’s electronic medical records. All-cause mortality data (date, if occurred) are automatically updated in the hospital records from Israeli’s social security agency *via* the Ministry of Health and can be retrieved by patient’s identification number. The study protocol was approved by the local institutional ethics committee which voided the need for obtaining informed consent.

### Study definitions

Electrocardiograms were recorded with a FUKUDA Cardimax 4 ECG machine and stored electronically. The QT using the Bazett formula is measured automatically. For quality assurance, QT interval measurements were validated by a senior electrophysiologist experienced in QT interval measurements, corroborating 4.1% of all QT interval measurements (203 of 4,904 total ECGs, each patient had to have at least 3 ECGs to be included in the study). For these manual measurements, the QT interval was measured using the “tangent” method. Briefly, a tangent was drawn to the steepest last limb of the presumed T wave to define the end of the T wave as the intersection of this tangent with the baseline. The validation showed high agreement between measurements, with a Pearson correlation = 0.94, [95% Confidence Interval (CI) = 0.925–0.956]).

Echocardiographic measurements were made using the same equipment for each examination (iE33, Philips Medical Systems, Bothell, WA, USA) by a specialized operator and in accordance with the published guidelines of the American Society of Echocardiography. Echocardiographic evaluations of left ventricular ejection fraction were interpreted by a specialized physician according to Simpson’s method. Infarct-related artery (IRA) was defined in the procedural report by the interventional cardiologist. Significant CAD was defined as stenosis of above 70% of the coronary lumen. Multi-vessel disease was defined as more than 1 vessel CAD with a narrowing of 50% or above.

### Statistical analysis

Missing numeric data was handled by imputation where its proportion did not exceed 30% and excluded otherwise. Missing categorical and outcome data were excluded. Associations were measured by linear and logistic regressions. Comparisons between groups were made by *T*-tests, Wilcoxon tests, ANOVA, and Kruskal–Wallis depending on the number of groups compared and the nature of the measurement. Mixed-effects models accounted for repeated measurements. Survival analysis was done by log-rank tests and Cox proportional hazards. Where multiple observations were available for each patient (such as QT from sequential ECGs), The Cox proportional hazard model was corrected to the mixed effects of the random. All analyses were performed in “R” version 4.1.2 [R Core Team (18). R: A language and environment for statistical computing. R Foundation for Statistical Computing, Vienna, Austria].^1^

## Results

### Study population

Our cohort included 5021 ECGs, taken on consecutive days from 1,054 patients hospitalized for STEMI. The median age was 62.22 (± 13.08) years, and 70.6% were males. IRA information was available for 883 patients. Baseline characteristics of the patients are presented in Table 1 and Supplementary Table 1 stratified by IRA. Generally, females in the cohort were older (65.2 ± 14 versus 60.7 ± 12.3 in males), had a slightly better renal function, and had higher rates of hypertension. No other clinical risk factors or characteristics varied between the genders.

**TABLE 1:** Baseline patient characteristics.

	Overall	Females	Males	*P*-value
Patients	1054*	290 (29.4)	698 (70.6)	–
Age [mean (SD)]	62.22 (13.08)	65.2 (14.3)	60.7 (12.2)	0
DM (true) (%)	279 (26.4)	177 (25.4)	87 (30)	0.14
HTN *n* (%)	479 (45.4)	153 (52.8)	294 (42.2)	0.002
EGFR [mean (SD)]	79.02 (25.42)	77.9 (23.6)	82.4 (29.2)	0.03
Hyperlipidemia *n* (%)	536 (50.9)	518 (49.1)	132 (45.5)	0.21
Family history (false) (%)	285 (27.0)	217 (75.3)	503 (72.1)	0.32
Smoker *n* (%)	513 (49.2)	513 (49.2)	125 (44.8)	0.09
Past MI *n* (%)	198 (18.8)	57 (19.7)	118 (16.9)	0.35
EF [mean (SD)]	46.01 (8.04)	45.51 (8.02)	46.1 (7.9)	0.42
All-cause mortality *n* (%)	68 (6.4)	28 (9.7)	39 (5.6)	0.029
**IRA (%)**				0.49
LAD	470 (53.2)	150 (55.4)	320 (52.3)	
LCX	121 (13.7)	32 (11.8)	89 (14.5)	
RCA	292 (33.1)	89 (32.8)	203 (33.2)	
Single vessel disease (%)	393 (44.5)	127 (46.9)	265 (43.4)	0.37
QT upon arrival [mean (SD)]	422.63 (33.3)	428.6 (33.4)	419.8 (32.5)	0

*The gender of 67 patients was not available.

### QT interval

The QT was considerably longer among females (428.64 ms, SD = 33.44 in females vs. 419.85 ms, SD = 32.52 in males, *P*-value = 0.001), this observation was consistent across all ECGs during hospitalization (*P* = 0.001). Age was a significant modifier of the QT interval: patients above the age of 65 years had a longer baseline QT (431 ms, SD = 37.4), compared with patients under 65 years (416 ms, SD = 28.4) (*P* = 0.0002). The baseline QT did not vary by the IRA, and was 421.34, SD = 34.89, 423.02, SD = 33.03, 421.47 ± 28.46 in the left anterior descending artery (LAD), and left circumflex artery (LCX), and the right coronary artery (RCA), respectively (*P*-value = 0.87).

### QT dynamics during hospitalization

**FIGURE 1:**
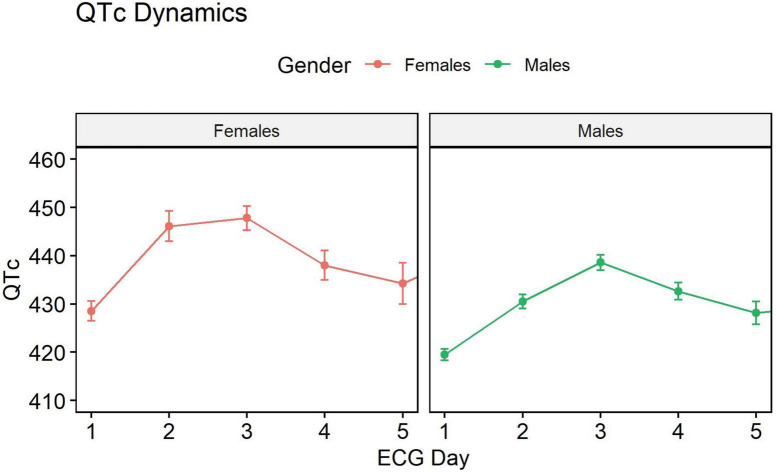
QT dynamics during hospitalization. QT interval prolongs during the initial 3 days past myocardial infarction (MI), and then starts shortening, but without returning to the baseline values.

QT upon arrival was 422 ms ±33. The QT interval prolongated by an average of 10 ms during the initial 3 days post-MI, and then started shortening back, albeit without returning to baseline values (Figure 1). The prolongation from baseline was 2.4 ms with each additional ECG (*P* = 0.0007) and was more prominent among females (9 ms increase *P* = 0.0005), patients with STEMI caused by occlusion of the LAD coronary artery (+5.3 ms compared with the RCA, *P* = 0.01), but not compared to the LCX artery (+3.6, *P* = 0.19). QT also increased more among patients older than 65 years (+16 ms, *P* = 0.0004).

### QT and clinical outcomes

A total of 68 (6.45%) patients died during a median follow-up period of 6 years [interquartile range (IQR) 2.11–4.54]. A total of 28 patients died within 1 month and 40 patients died during the long-term follow-up.

**FIGURE 2:**
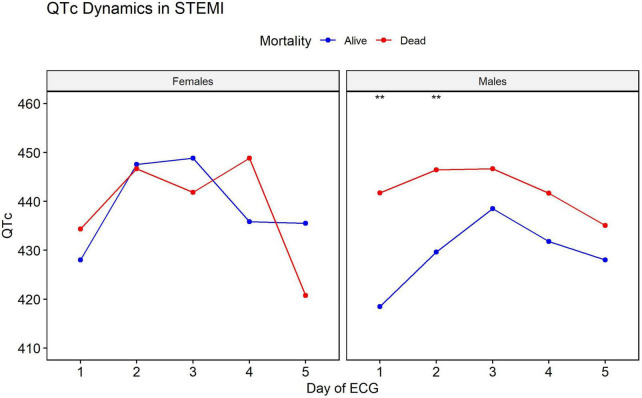
Difference in QT interval dynamic between men and women. In men, consistently longer QT intervals were associated with mortality. This dynamic and associated mortality difference is not seen in women. **Statistical significance with a *P*-value of <0.01.

The median ECG follow-up time was 3 days (IQR 2–4 days). The QT interval at presentation, as well as the average QT values throughout hospitalization, predicted time to all-cause mortality. Older age was associated with higher mortality rates in the presence of longer QT intervals (*P*-value = 0.0002). Among males, consistently longer QT intervals were associated with mortality. This dynamic and associated mortality difference was not observed among females (Figure 2).

**FIGURE 3:**
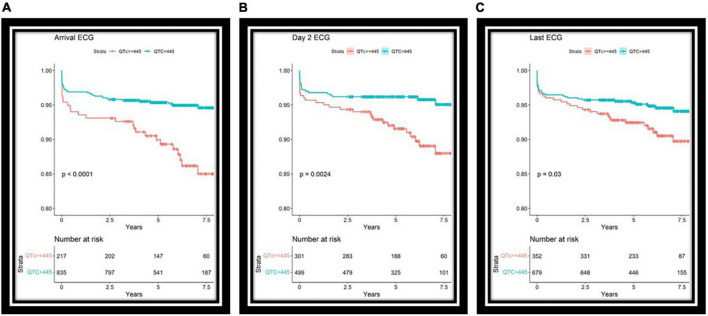
Survival curves for long or normal QT groups based on ECGs upon arrival **(A)**, day two of hospitalization **(B)**, and the ECG before discharge **(C)**. Interestingly, longer QT intervals are associated mainly with long-term mortality and may reflect overall health. Lower panel, number of patients at risk each day.

The long QT group had increased mortality in univariate cox analysis on the first, the second, and on the day of discharge [HR 2.54, CI (1.56–4.13), HR 2.32, CI (1.32–4.07), and HR 1.69, CI (1.04–2.75)] (Figures 3A–C).

**FIGURE 4:**
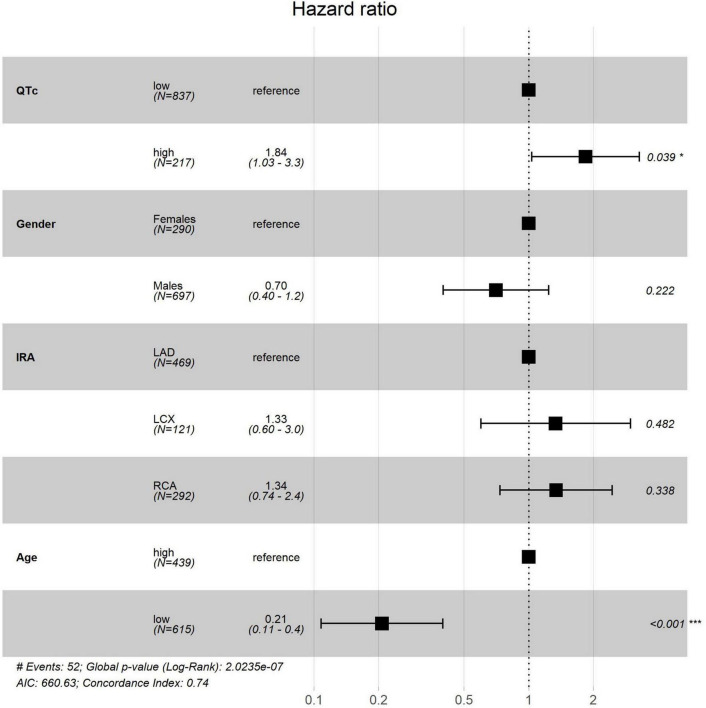
Multivariate Cox analysis. HR, hazard ratio; CI, confidence interval; IRA, infarcted related artery; LCX, left circumflex; RCA, right coronary artery. *Based on ECG upon arrival. ***Statistical significance, with a *P*-value <0.001.

In a multivariate model which included gender, age, and the IRA, QT on arrival was independently associated with increased mortality (*P* = 0.01) (Figure 4).

Other data regarding QT prolongation is provided in Supplementary Table 2.

## Discussion

Our study indicates that prolonged QT is strongly and independently associated with mortality among patients with STEMI, providing incremental information to other known clinical risk factors.

Published data demonstrated the association between a prolonged QT interval and all-cause mortality in the general population (2, 3). Ventricular repolarization may delay due to myocardial ischemia or stress, prolonging of the QT interval, and predisposing to sudden death (7, 8). A prolonged QT interval is associated with autonomic dysfunction and hyperactivity that may be manifested as elevated blood pressure, atherosclerosis, and cardiovascular events (7–10).

We characterized the QT behavior during the immediate period following acute STEMI. The QT interval elongates starting at the acute event and peaks at day three when it begins to shorten. At day five, which serves as a common discharge timepoint, QT does not reach baseline values. Prolonged QT duration at hospitalization, at day two, and prior to discharge is associated with mortality in males, but not in females (with the caveat that the smaller number of females in our cohort may have hampered the power to detect such associations).

Our large sample size and multiple ECGs allowed characterizing a more significant effect size of prolonged QT than previously reported. The establishment of the days in which QT values can identify higher-risk patients is clinically relevant and may aid in the decision-making process of which patients can be discharged early, and which patients may benefit from longer observation, a common clinical dilemma.

An interesting point is that the QT in the setting of STEMI was predictive of long-term mortality. This is represented by the fact that out of the 68 deaths in our cohort, 28 occurred more than a year after the acute event, 22 occurred more than 2 years and 17 more than 3 years following the episode. This may imply that the tendency to prolong QT in STEMI may reflect an abnormal repolarization reserve, which is a marker of poor health.

## Limitations

A retrospective study is subject to the known inherent biases of its design. We excluded 3,882 patients due to missing crucial information (at least three ECG recordings during the index hospitalization), which could be the source of chart selection and bias. In addition, our database had no information regarding the cause of death, medications, or serum electrolytes, which are important for any discussion regarding the QT interval. Finally, the current results are registry-based, in which angiographic data were collected prospectively based on the interpretation of the interventional cardiologist during the index admission and were not reviewed by an independent committee.

## Conclusion

QT interval elongates during STEMI, this prolongation is associated with increased mortality. Gender is an important mediator of QT dynamics.

## Data availability statement

The original contributions presented in this study are included in the article/Supplementary material, further inquiries can be directed to the corresponding author.

## Ethics statement

The studies involving human and animal participants were reviewed and approved by the Tel Aviv Medical Center Ethics Committee.

## Author contributions

All authors listed have made a substantial, direct, and intellectual contribution to the work, and approved it for publication.

## Footnotes

1
https://www.R-project.org/
