# Supplementary material for: QT interval dynamics in patients with ST-elevation MI
Source: Front Cardiovasc Med. 2023 Jan 6;9:1056456. doi: 10.3389/fcvm.2022.1056456 (PMC9853398; doi:10.3389/fcvm.2022.1056456)
Supplement: Supplementary file 1 [file Data_Sheet_1.docx]

Figure S1: Consort plot


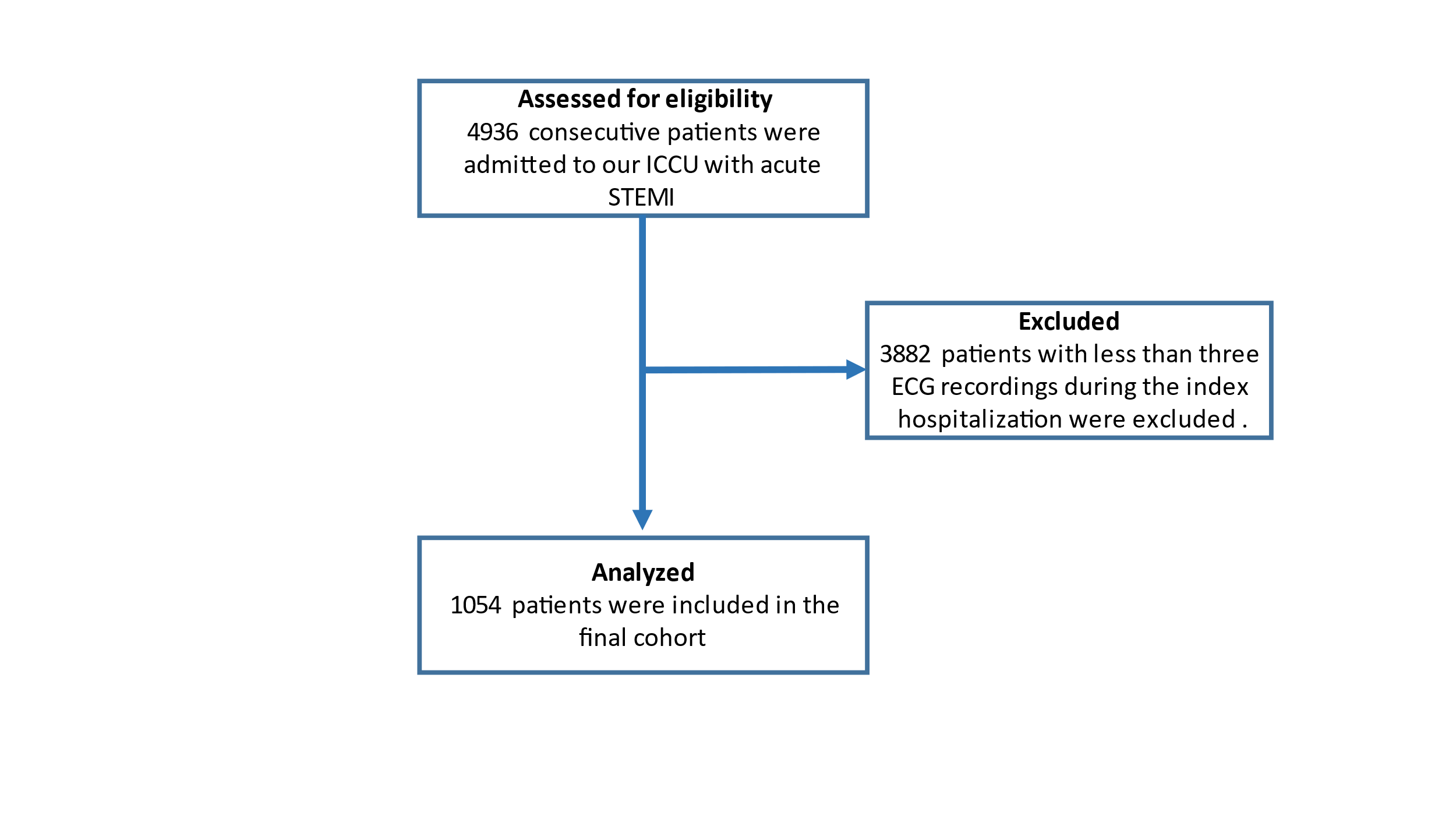


Table S1: Patient characteristics by the Infarct Related Artery

| **P** | **RCA** | **LCX** | **LAD** |  |
| --- | --- | --- | --- | --- |
|  | 292 | 121 | 470 | Number of Patients |
| 0.132 | 83 (28.4) | 38 (31.4) | 111 (23.6) | DM (%) |
| 0.32 | 80.19 (26.19) | 83.07 (28.36) | 79.17 (24.05) | EGFR (mean (SD)) |
| 0.889 | 61.75 (12.00) | 61.17 (13.19) | 61.38 (13.26) | Age (mean (SD)) |
| 0.204 | 155(53.1) | 63 (52.1) | 220 (46.8) | Hyperlipidemia (%) |
| 0.151 | 155 (53.4) | 65 (54.2) | 217 (47.1) | Smoker (%) |
| 0.339 | 137 (46.9) | 56 (46.7) | 197 (41.9) | HTN (%) |
| 0.578 | 51 (17.5) | 20 (16.5) | 69 (14.7) | Past Mi (%) |
| <0.001 | 49.17 (6.97) | 48.62 (6.03) | 43.46 (7.77) | EF (mean (SD)) |
| 0.071 | 407.01 (34.93) | 405.21 (44.81) | 399.89 (46.53) | Minimum QTc (mean (SD)) |
| <0.001 | 446.33 (30.38) | 452.26 (41.02) | 463.54 (37.52) | Maximum QTc (mean (SD)) |
| <0.001 | 39.32 (37.27) | 47.06 (52.22) | 63.65 (48.00) | Delta (mean (SD)) |
| <0.001 | -1.08 (0.70) | -0.97 (0.67) | -0.72 (0.65) | Qt Variability Index (mean (SD)) |
| 0.506 | 203 (69.5) | 89 (73.6) | 320 (68.1) | Gender (Male %) |
| <0.001 |  |  |  | I.R.A (%) |
|  | 0 (0.0) | 0 (0.0) | 470 (100.0) | LAD |
|  | 0 (0.0) | 121 (100.0) | 0 (0.0) | LCX |
|  | 292 (100.0) | 0 (0.0) | 0 (0.0) | RCA |
| <0.001 | 194 (66.4) | 70.3 (60.3) | 223 (47.4) | Multivessel Disease (%) |
| 0.877 | 421.47 (28.46) | 423.02 (33.03) | 421.34 (34.89) | QTc at Arrival (mean (SD)) |

Table S2: clinical and outcome characteristics of patients with and without QT prolongation*

| **P value** | **QT not prolonged** | **QT prolonged** | **Parameter** |
| --- | --- | --- | --- |
| 0.006 | 63.05 (13.41) | 60.63 (12.45) | Age (mean (SD)) |
| 0.147 | 200 (78.2) | 428 (82.8) | Gender (Male %) |
| <0.001 | 43.85 (7.21) | 47.46 (7.77) | EF (mean (SD)) |
| <0.001 | 52 (16.0) | 50 (8.1) | HF (%) |
| 0.14 | 78 (23.9) | 177 (28.6) | DM (%) |
| 0.705 | 160 (49.7) | 312 (51.2) | smoker (%) |
| 0.231 | 153 (46.9) | 263 (42.6) | HTN (%) |
| 0.874 | 165 (50.6) | 308 (49.8) | Hyperlipidemia (%) |
| 0.547 | 10 (4.3) | 14 (3.1) | Died (%) |

*QT prolongation is defined as being above/below the mean of maximal QT to minimal QT difference.
